# Clinical characteristics and surgical outcomes of transcutaneous versus transconjunctival excision of Wolfring gland ductal cysts

**DOI:** 10.1186/s12886-024-03420-x

**Published:** 2024-04-16

**Authors:** Mostafa Mohamed Diab, Richard C. Allen, Khaled Kotb Mohammed, Ahmed T.S. Saif

**Affiliations:** 1 Dept. of Ophthalmology, Faculty of Medicine, Fayoum University, 6 Elnabawy St, Borg Al Atebbaa, Al Mesalla, 63514 Al Fayoum, Egypt; 2 Dept. of Ophthalmology, Dell Medical School, University of Texas at Austin, Texas Oculoplastics Consultants, Austin, TX USA

## Abstract

**Purpose:**

To analyze the clinicopathological characteristics and surgical outcomes of patients with Wolfring gland ductal cysts (WGDCs).

**Methods:**

A retrospective, consecutive, interventional comparative case series was performed over a period of 7 years. Data on demographic and clinical characteristics, pathological findings and outcomes of surgically excised cysts were collected. A comparison between the transconjunctival and transcutaneous approaches was also assessed.

**Results:**

Forty-eight patients (48 eyelids) were included in the final analysis. The most common presenting symptom was painless eyelid swelling (81.3%). The median symptom duration was 11.5 months (IQR, 18.25). The upper eyelid was involved in 31 (64.6%) patients, 29/31 of whom had cysts in a medial or centromedial location. Forty-five (93.8%) cysts were bluish gray and transilluminable with clear contents on lid eversion and a median largest dimension of 22 mm (IQR, 8). A transverse conjunctival fibrotic band was observed along the proximal tarsal border in the cyst area in all patients. Signs of chronic trachoma were noted in 38 (79.2%) patients. Preoperative significant ptosis was present in 28/31 (90.3%) of the upper eyelid cysts. Thirty cysts (62.5%) were excised through the skin, and 18 cysts (37.5%) were excised transconjunctivally. Intraoperative cyst rupture, the need for conjunctival grafting and postoperative residual upper lid ptosis were significantly greater in the transconjunctival group (*p* = 0.009, *p* < 0.001, and *p* = 0.016, respectively).

**Conclusion:**

The present study highlights the clinicopathological characteristics of a relatively large series of surgically excised WGDCs. Transcutaneous excision of WGDCs has proven to be an effective treatment with fewer adverse sequelae than the transconjunctival approach.

**Supplementary Information:**

The online version contains supplementary material available at 10.1186/s12886-024-03420-x.

## Introduction

Wolfring gland ductal cysts (WGDCs) are cystic dilatations of the ducts of the accessory lacrimal glands of Wolfring that are embedded in the deep conjunctival stroma along the proximal tarsal border [1]. Their nomenclature includes the following: Wolfring dacryops, ductal cysts of accessory lacrimal glands of Wolfring, cysts of the accessory lacrimal glands of Wolfring [2], Wolfring ductal cysts, and accessory lacrimal gland ductal cysts [3]. The pathogenesis of WGDC development is still debatable and likely multifactorial. Conditions causing chronic conjunctival inflammation and subconjunctival scarring, such as trachoma, ocular cicatricial pemphigoid (OCP), Stevens-Johnson syndrome (SJS), trauma or chemical injury, have been associated with dacryops formation [1, 4–7].

A few case series and reports have been published on WGDCs [8–11]. This may be due to their relative rarity in Western countries or high rates of misdiagnosis in countries where they may be more prevalent [11].

Various treatment modalities, such as excision, simple aspiration, marsupialization, or needling, have been described for dacryops [1, 3, 12, 13]. Complete surgical removal remains the mainstay treatment and can be performed either transconjunctivally or transcutaneously, with no proven benefit between these 2 surgical approaches [1, 3, 14].

In this study, we analyzed the presenting features, pathological characteristics, and intraoperative findings and compared the postoperative outcomes of patients who underwent excision of WGDCs either through the skin or transconjunctivally.

## Subjects and methods

A retrospective chart review was conducted between May 2015 and June 2023 at the Department of Ophthalmology, Faculty of Medicine, Fayoum University, Al Fayoum, Egypt; 48 patients (48 eyes) were identified who underwent excision of WGDCs under the care of the first author. The diagnosis of WGDC was based on clinical presentation (a subconjunctival cyst associated with the proximal tarsal border) and histopathological confirmation (a nonkeratinizing epithelial cyst with a double-layer lining and a fibrous wall). All patient information was deidentified once the data collection process was complete. Informed consent was obtained from patients whose photographs were considered for publication. This study was approved by the Fayoum University Faculty of Medicine Institutional Review Board (IRB) prior to data collection. The study was conducted in accordance with the principles of the Declaration of Helsinki.

The collected data included demographic information, the type and duration of symptoms, clinical features of the cyst (i.e., location, cyst size, color, tenderness, relation to the proximal tarsal border, related conjunctival scarring and/or symblepharon, signs of chronic trachoma, prior treatments, secondary eyelid malpositions, and orbital signs), surgical technique (transcutaneous or transconjunctival), intraoperative findings (color, tarsal attachment, rupture, conjunctival grafting), histopathological data (the epithelial lining, the presence of goblet cells, inflammatory cells or glandular tissue), postoperative outcomes (scar visibility, eyelid malposition, and recurrence), and follow-up duration.

The eyelids were completely everted to allow adequate visibility and measurement of the cyst size. In cases where complete eversion of the eyelid was difficult, measurements were taken intraoperatively following full exposure of the anterior surface of the cyst. The palpebral conjunctiva was examined for signs of chronic trachoma (tarsal conjunctival scarring, posttrachomatous degeneration, limbal pannus, or corneal scarring) or any scarring in the area of the cyst.

Scar visibility was assessed with eyes open at a 1 m distance by an independent oculoplastic fellow. If it was not possible to detect the side of surgery, the scar was assessed as invisible. If the scar was noticeable, it was graded as minimally visible, moderately visible, or easily visible.

A comparison between the tranconjunctival and transcutaneous approaches regarding intraoperative cyst rupture, the need for conjunctival grafting, postoperative lid malpositions, recurrence, and scar visibility was also performed.

### Surgical technique

#### Transcutaneous approach

##### Upper eyelid

**Fig. 1:**
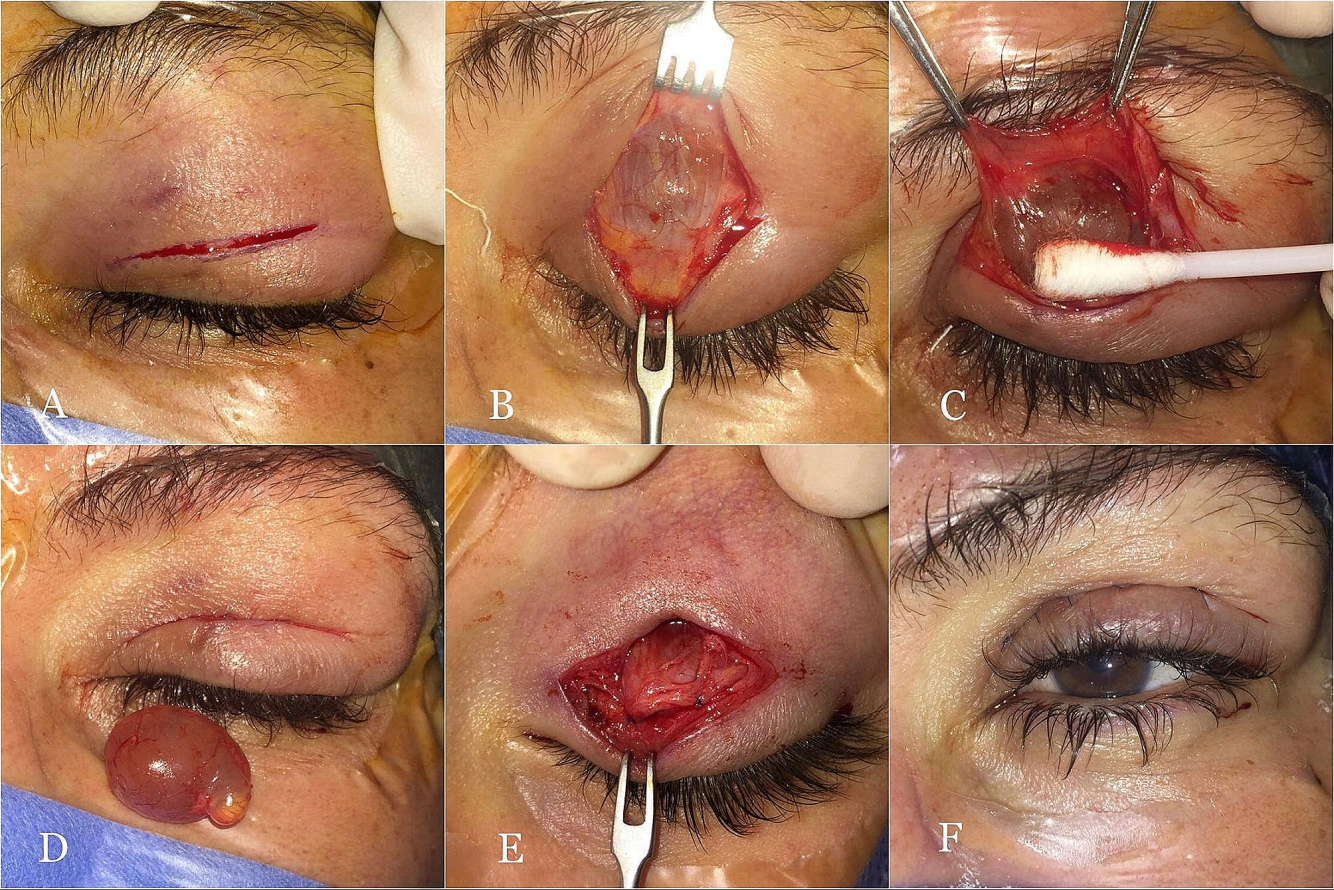
Transcutaneous approach for upper eyelid Wolfring gland ductal cysts. (**A**) Medial eyelid crease incision. (**B**) The cyst appears beneath the levator aponeurosis after opening the orbital septum and retracting the preaoneurotic fat. (**C**) The levator Muller complex is dissected from the anterior surface of the cyst with the aid of a cotton swab. (**D**) The cyst is completely excised with an intact wall. (**E**) The levator aponeurosis is reattached to the tarsal plate. (**F**) After skin closure

Through a skin crease incision (Fig. 1A), the orbital septum was opened to expose the anterior surface of the levator aponeurosis in the area of the cyst (Fig. 1B). Using Wescott scissors, the levator aponeurosis was disinserted from the anterior surface of the tarsus and dissected from the underlying Mueller muscle a few millimeters. Just superior to the peripheral vascular arcade, the Mueller muscle was transected and separated from the underlying cyst with the aid of cotton-tipped applicators, exposing the whole anterior surface of the cyst (Fig. 1C). Dissection was then continued between the cyst and the conjunctiva craniocaudally until the area of dense fibrotic adhesion was observed along the proximal tarsal border. Careful sharp dissection was then required to detach the cyst from the superior tarsal border aiming for intact cyst removal (Fig. 1D). If small conjunctival buttonholesoccured, they were not sutured. Following cyst excision, the levator aponeurosis was reattached to the tarsus, followed by skin closure (Fig. 1E, F).

##### **Lower eyelid** (supplementary fig. S1A-F)

Through an infraciliary incision, the orbicularis muscle was transected while preserving the pretarsal segment. The lower eyelid retractor was then detached from the inferior tarsal border to expose the anterior surface of the cyst. Gentle dissection was performed to separate the cyst from the surrounding tissues with the aid of cotton swabs until the area of tarsal attachment, where sharp dissection was needed.

##### Transconjunctival approach (Fig. 2A-H)

Limbal traction and eyelid margin sutures were placed to enhance surgical exposure. An incision was made through the conjunctiva to one side of the cyst away from the area of dense fibrous adhesion along the proximal tarsal border. A subconjunctival plane over the surface of the cyst was created. With the aid of cotton swabs, dissection was continued around the cyst, separating it from the surrounding tissues. At the area of the tarsal attachment, gentle sharp dissection was required to fully separate the cyst. The conjunctival defect was assessed for primary closure or grafting.

**Fig. 2:**
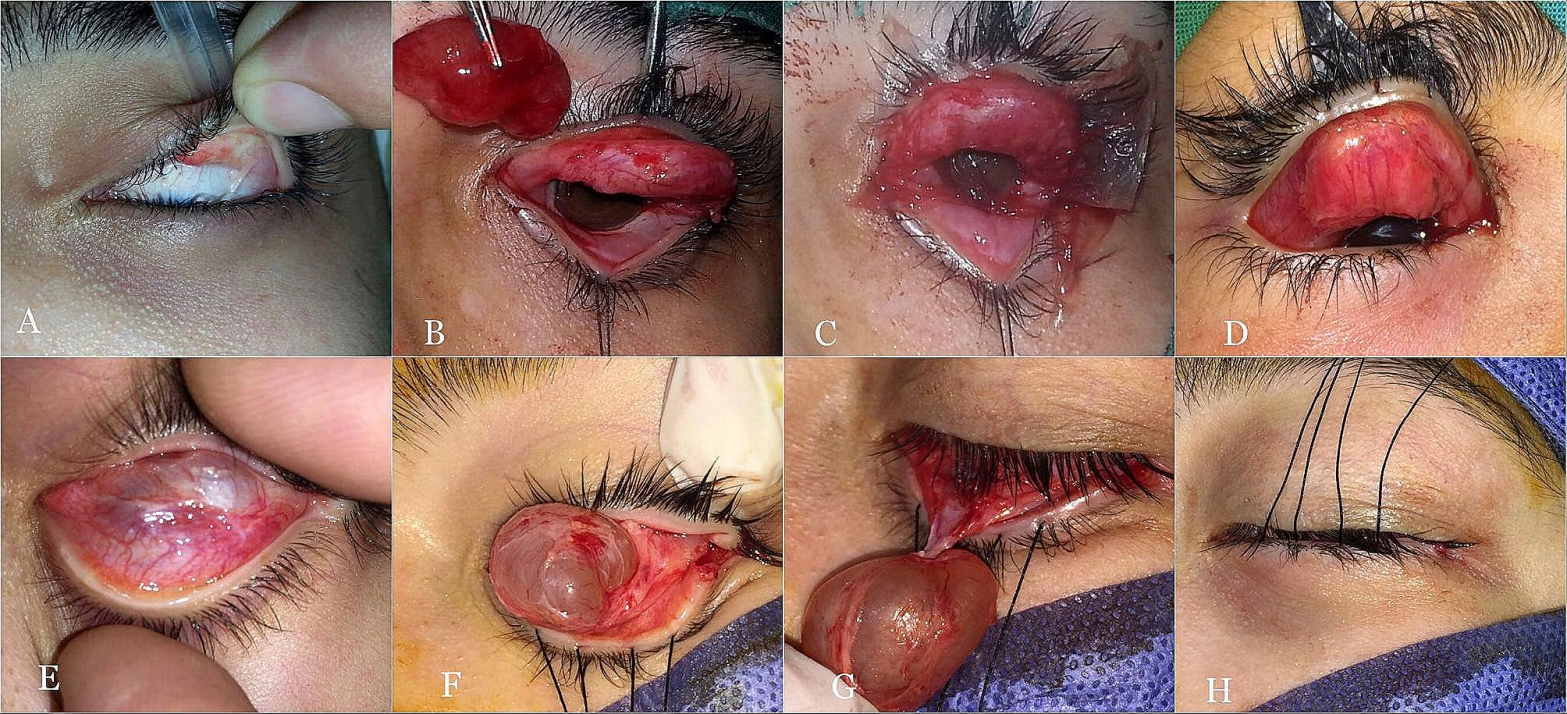
Transconjunctival approach for upper and lower Wolfring gland ductal cysts. (**A**) The upper eyelid is everted with difficulty due to conjunctival scarring. (**B**) A large cyst with an intact wall was removed, leaving a large conjunctival defect. (**C**) An amniotic membrane graft was used to cover the defect. (**D**) A conjunctival autograft is sutured in place in another patient. E A cyst is visible through the conjunctiva of an everted lower eyelid. (**F**, **G**) The cyst is dissected from the surrounding tissues away from the area of fibrotic adhesions first. **H** At the end of the surgery

### Statistical analysis

The statistical analysis was performed using SPSS version 27 (IBM, Inc.) for Windows. Numerical data are presented as means and standard deviations if normally distributed or as medians and IQRs if not normally distributed, and categorical variables are presented as numbers and percentages. The level of significance was < 0.05. The chi-square test or Fisher’s exact test was used to analyze categorical data, and the Mann‒Whitney test was used to analyze numerical data. Numerical data were compared using an independent sample t test if normally distributed or the Mann‒Whitney U test if not normally distributed.

## Results

### Demographic features

A total of 48 patients (31 female; 64.6%) with WGDC were surgically treated over a 7-year period. The mean age at presentation was 28.5 years (SD 14.6; range 3–66 years). All the cases were unilateral.

Thirty-nine patients (81.3%) presented with a painless eyelid mass. Nine patients (18.8%) reported pain and tenderness to touch. The mean duration of symptom(s) was 16.4 months (median 11.5 months; range 4–56). Most (45/48) patients had an insidious course; however, 3 patients experienced rapid growth over a few days with subsequent complete ptosis.

Four patients (8.3%) had a history of vernal keratoconjunctivitis (VKC) during childhood. Six patients (12.5%) underwent simple aspiration or needling of the cyst, followed by recurrence. None of the patients had a previous history of trauma.

### Clinical features of WGDCs

**Fig. 3:**
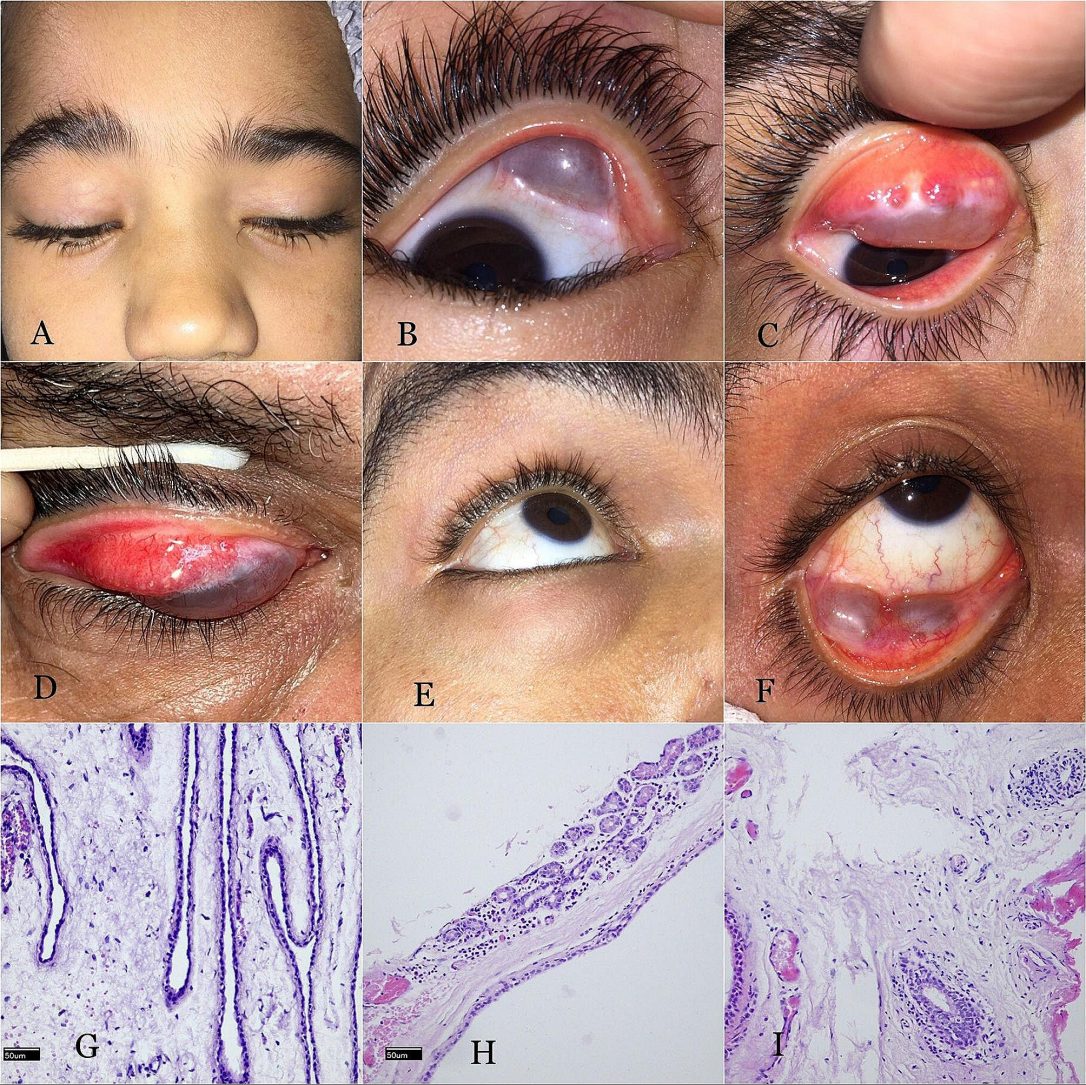
Typical clinical and histologic features of Wolfring gland ductal cysts. (**A**) A mass in the right upper lid medially of a 6-year-old child. (**B**) A bluish gray cystic lesion in the superomedial fornix of the right eye of the same patient. (**C**) A dense fibrotic band along the proximal tarsal border with complete lid eversion. (**D**) Signs of trachoma adjacent to a WGDC in the upper eyelid of another patient. (**E**) A mass in the right lower eyelid centrally of a 34-year-old man. (**F**) The lesion is visible through the conjunctiva as a bluish gray cystic mass with surrounding scarring and symblephara. (**G**) A cyst is lined by a double-layered nonkeratinizing cuboidal epithelium with a serpiginous cavity (original magnification, ×200). (**H**) Adjacent lacrimal glandular tissue. (**I**) Periductular chronic inflammatory cells

The upper eyelid was involved in 31 (64.6%) patients; 29/31 of the cysts had a medial or centromedial location, and 2/31 involved the entire lid. In the lower lid, 6/17 of the cysts were central, 6/17 were medial, and 5/17 were lateral. A mass was noticeable through the skin in all patients (Fig. 3A, E).

On eyelid eversion, 45 (93.8%) cysts appeared bluish gray and transilluminable with clear coloration (Fig. 3B, C, D, F), 2 (4.2%) cysts had yellowish opaque material with surrounding conjunctival inflammation (Supplementary Fig. S2A, B), and 1 (2.1%) cyst was chocolate colored (Supplementary Fig. S2C, D). All the cysts were smooth and walled, with a median largest diameter of 22 mm (IQR, 8.5; range, 12–45 mm) (Supplementary Fig. S2E, F).

There was a transverse, fibrotic band over the cyst along the proximal tarsal border in the area of the cyst in all patients (Fig. 1C, D, F). Signs of chronic trachoma (palpebral conjunctival scarring, posttrachomatous degenerations, pannus siccas, or corneal opacity) were present in 38 (79.2%) patients (Fig. 1D). Conjunctival fibrosis was observed in the upper tarsal conjunctiva in the 4 patients with a history of VKC. Ptosis was present in 28/31 (90.3%) of the upper eyelid cysts. Globe dystopia was found in 9 (18.8%) patients. There was no mechanical restriction of ocular motility or diplopia.

### Pathologic features

Intact cysts appeared smooth and walled with telangiectatic blood vessels on the surface.

Forty-five cysts (93.8%) were filled with clear fluid, 2 (4.2%) had yellow mucopurulent material, and 1 (2.1%) contained blood.

Histologic examination revealed a double-layered nonkeratinizing epithelial cyst in all the examined specimens, with a serpinginous cavity in 43 (89.6%) cysts (Fig. 1G). Lacrimal glandular acini were present in 12 (25%) (Fig. 1H), inflammatory cells in 38 (79.2%) (Fig. 1I) and goblet cells in 40 (83.3%) cysts. Table 1 details the demographic data and clinicopathologic features of the WGDC patients (Table 1).

**Table 1:** Clinical and histological characteristics of the study subjects

Variable		Total (*N* = 48)	Transcutaneous (*N* = 30)	Transconjunctival (*N* = 18)	P-value
Age, mean (SD)		28.54(14.59)	29.33 (14.49)	27.22 (15.08)	0.633
Gender, female, N (%)		31 (64.6%)	20 (66.7%)	11 (61.1%)	0.697
Eyelid, upper, N (%)		31 (64.6%)	19 (63.3%)	12 (66.7%)	0.815
Location	Medial	33 (68.8%)	17 (56.7%)	16 (88.9%)	0.119
	Central	8 (16.7%)	7 (23.3%)	1 (5.6%)	
	Lateral	5 (10.4%)	4 (13.3%)	1 (5.6%)	
	Whole lid	2 (4.2%)	2 (6.7%)	0 (0%)	
Duration of symptom (s) in months, median (IQR)		11.5 (18.25)	12.5 (19)	10.5 (17.5)	0.823
History of recurrence		2 (4.2%)	0 (0%)	2 (11.1%)	0.136
Color	Bluish	45 (93.8%)	28 (93.3%)	17 (94.4%)	0.287
	Yellowish	2 (4.2%)	2 (6.7%)	0 (0%)	
	Hemorrhagic	1 (2.1%)	0 (0%)	1 (5.6%)	
Reaching the proximal trasal border		48 (100%)	30 (100%)	18 (100%)	-
Tenderness		9 (18.8%)	6 (20%)	3 (16.7%)	> 0.999
Largest dimension in mm, median (IQR)		21 (8)	20 (8)	22(8)	0.204
Fibrotic band along the proximal tarsal border, N (%)		48 (100%)	30 (100%)	18 (100%)	-
Symblepharon		31 (64.6%)	20 (66.7%)	11 (61.1%)	0.697
Signs of trachoma		38 (79.2%)	24 (80%)	14 (77.8%)	> 0.999
Ptosis in upper lid cases, proportion (%)		28/31(90.3%)	18/19 (94.7%)	10/12 (83.3%)	0.543
Orbital signs (dystopia/proptosis), N (%)		9 (18.8%)	3 (10%)	6 (33.3%)	0.063
Histopathologic features					
Goblet cells, N (%)		40 (83.3%)	27 (90%)	13 (72.2%)	0.229
Glandular acini, N (%)		12 (25%)	7 (23.3%)	5 (27.8%)	> 0.999
Inflammatory cells, N (%)		38 (79.2%)	22 (73.3%)	16 (88.9%)	0.282

## Intraoperative findings

The most common indication for excision was the presence of a mass (66.7%), followed by poor cosmesis (18.8%) and pain or chronic irritation (14.6%).

Intraoperatively, all cysts were found in the subconjunctival space and were firmly adherent to the proximal tarsal border.

Intraoperative cyst rupture occurred in 9 patients (18.8%). A large diameter of the cyst, recurrent cysts, and the use of the transconjunctival approach were associated with a greater risk of iatrogenic rupture (*p* = 0.031, *p* = 0.032, and *p* = 0.009, respectively). Trachomatous scarring was present in all patients with cyst rupture, although not to a statistically significant degree when compared with noncyst rupture (*p* = 0.661). Two patients (2/9) with intraoperative rupture developed recurrent cysts during the follow-up period, for which the median duration was 36 months (interquartile range (IQR), 42.25; range, 6–120 months).

Reconstructive conjunctivoplasty using amniotic membrane transplantation or conjunctival autografting was performed in 7 patients (14.6%), all of whom underwent a transconjunctival approach (Fig. 2D, E).

### Outcomes

**Fig. 4:**
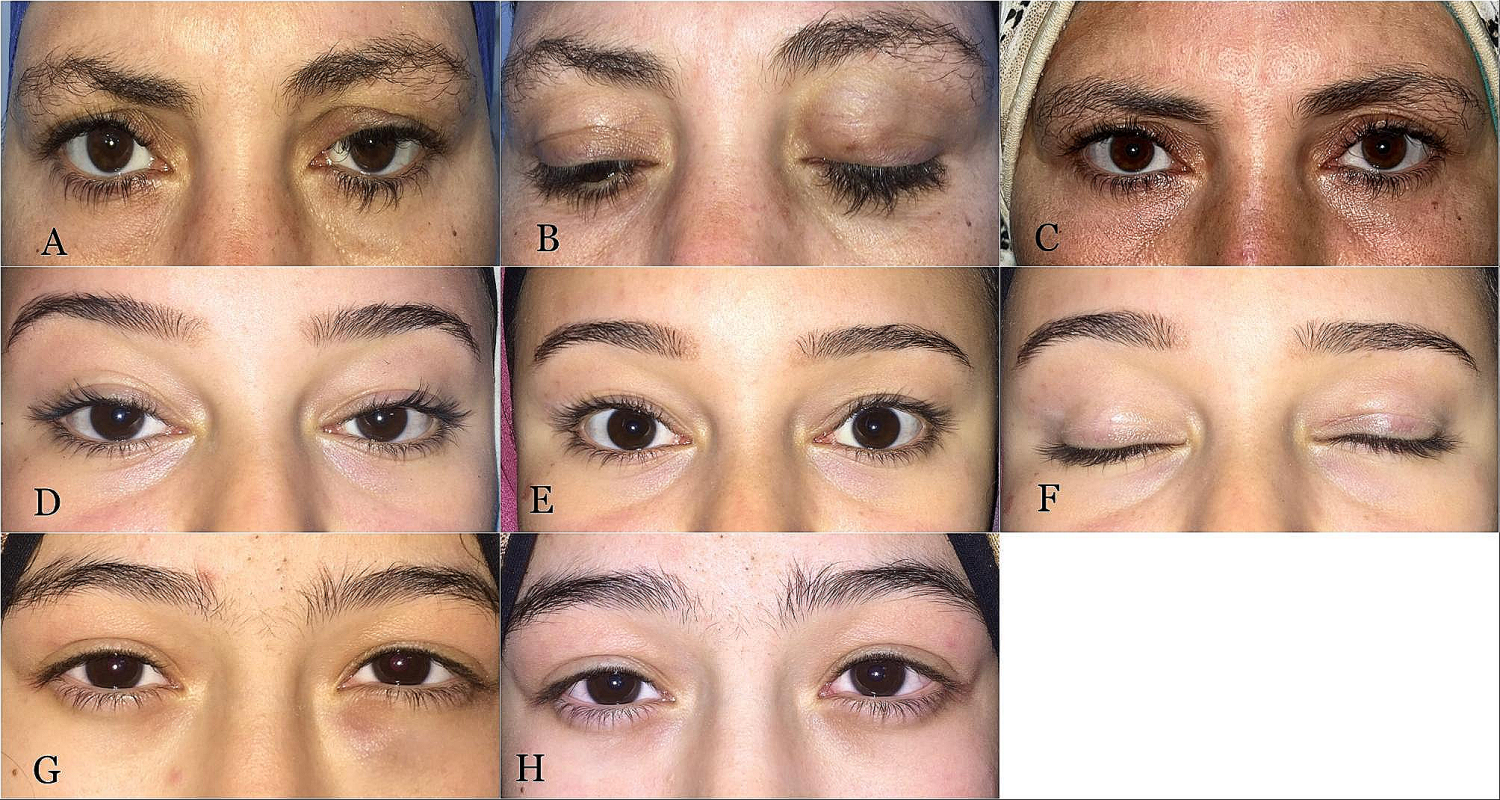
Pre-and postoperative clinical photographs. (**A**, **B**) Preoperative photographs of a 45-year-old woman with a left upper lid Wolfring gland ductal cyst medially and significant ptosis. (**C**) Postoperative appearance after transcuatneous cyst removal. (**D**) Preoerative appearance of a 21-year-old female patient with a left upper eyelid WGDC. (**E**, **F**) Postoperative images following transcuatneous cyst removal showing normal eyelid position without lagophthalmos. **G** Preoperative image of an 18-year-old girl with a left lower lid WGDC. **H** Postoperative appearance following transconjunctival cyst removal

In all patients who underwent surgery through the skin, the scar was graded as invisible. There were no cases of postoperative lagophthalmos or increased dry eye symptoms (Fig. 4).

**Fig. 5:**
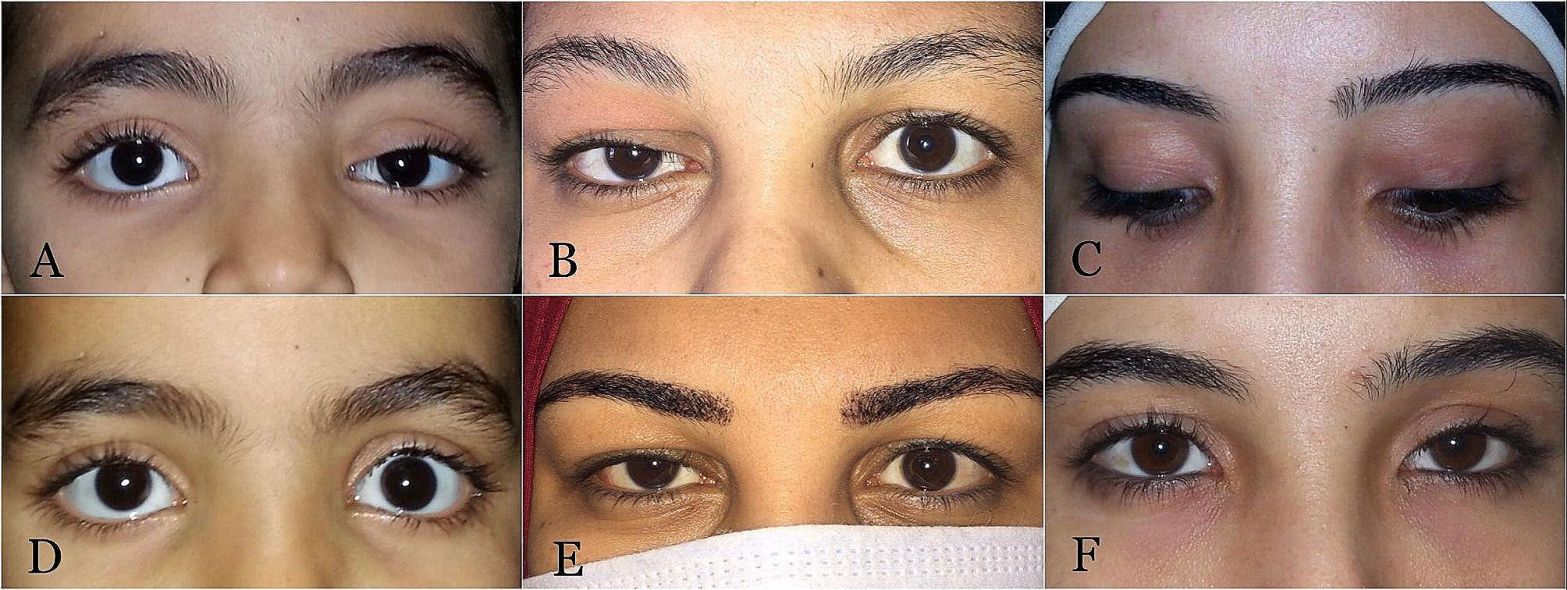
Postoperative adverse outcomes. (**A**) Preoperative image of a 6-year-old girl with left upper eyelid WGDC causing mechanical ptosis with inferior globe dystopia. (**B**, **C**) Preoperative images of 2 female patients with upper eyelid WGDCs. (**D**) Postoperative appearance of Patient A with eyelid retraction following transcuatneous cyst removal. Her condition improved with downward eyelid massage. (**E**, **F**) Postoperative images of patients B and C with residual ptosis following transconjunctival cyst removal

Four upper lid patients (4/31) had postoperative residual ptosis; all of whom underwent surgery transconjunctivally. Postoperative upper lid retraction occurred in two patients (2/31) following the transcutaneous approach. Both patients improved with downward eyelid massage (Fig. 5).

### Transcutaneous vs. transconjunctival approach

Intraoperative cyst rupture, the need for conjunctival grafting and postoperative residual upper lid ptosis were significantly greater in the transconjunctival group (*p* = 0.009, *p* < 0.001, and *p* = 0.016, respectively). There were no significant differences between the two groups regarding age, sex, eyelid status, duration of symptoms, associated trachoma, or largest cyst dimension (*p* = 0.633, *p* = 0.697, *p* = 0.815, *p* = 0.823, *p* > 0.999, *p* = 204, respectively) (Table 2).

**Table 2:** Intraoperative findings and postoperative outcomes in the study subjects

Variable		Total	Transcutaneous	Transconjunctival	P-value
Intraoperative findings					
Tarsal fixation		48 (100%)	30 (100%)	18 (100%)	-
Appearance, N (%)	Opaque	2 (4.2%)	1 (3.3%)	1 (5.6%)	> 0.999
	Translucent	46(95.8%)	29 (96.7%)	17 (94.4%)	
Cyst rupture, N (%)		9(18.8%)	2 (6.7%)	7 (38.9%)	0.009*
Conjunctival grafting, N (%)		7 (14.6%)	0 (0%)	7 (38.9%)	< 0.001*
Postoperative outcomes					
Residual upper lid ptosis, proportion (%)		4/31 (12.9%)	0/19 (0%)	4/12 (33.3%)	0.016*
Eyelid retraction, N (%)		2 (4.2%)	1 (3.3%)	1 (5.6%)	> 0.999
Scar visibility, Invisible, N, (%)		48 (100%)	30 (100%)	18 (100%)	-
Follow up period in months, median (IQR)		36 (42.25)	34 (40.5)	43.5 (42.75)	0.216

## Discussion

WGDCs have received little attention in published literature, and data on surgical outcomes are scarce. Since Weatherhead reported his case series of Wolfring dacryops in 1991 [1], only few case reports have addressed this topic [2, 12, 15, 16]. Other case series included several types of dacryops without a specific characterization of WGDCs. To the best of our knowledge, this is the largest case series describing the characteristics and surgical outcomes of WGDCs.

WGDC has been reported to be associated with conjunctival cicatrization, particularly trachoma, but the exact etiology has not been determined [1, 3]. In the present study, palpebral conjunctival scarring was present in 87.5% of the patients (79.2% due to trachoma and 8.3% due to VKC). Galindo-Ferreiro et al. reported that up to 85% of dacryops have been linked to earlier conjunctival scarring based on a review of the literature [3]. Trachomatous scarring was present in all the patients in Weatherhead’s series [1].

This association could explain the relative rarity of dacryops in Western countries; Salam et al. reported only 10 patients in an 18-year period in the UK [13]. Von Holstein et al.17 reported 24 patients in a 33-year period in Denmark [17], and Lam et al. identified 5 patients with dacryops over a 3-year period in the USA [18]. Most large series of accessory lacrimal gland cysts were reported in Saudi Arabia, previously endemic for trachoma [1, 3, 11].

Cicatrization seems to play a key role in the pathogenesis of dacryops [5, 19]. Our findings of the presence of fibrotic bands along the proximal tarsal border in all patients, palpebral conjunctival scarring in most patients and chronic inflammatory cells observed in most specimens may support this mechanism. Previous reports of accessory lacrimal ductal cysts in the context of conjunctival scarring also support this theory [1, 4, 15].

However, the role of cicatricial ductular blockage as the sole factor in dacryops formation has been challenged experimentally [9]. Some authors propose concurrent IgA hypersecretion by activated plasma cells with a resultant osmotic effect [1, 18]. Others have suggested dysfunction of the periductular neural plexus [5].

The presence of lacrimal glandular tissue next to the cyst is considered the main histologic clue to lacrimal gland (LG) dacryops [5]. However, this diagnostic feature was not universally present in most patients in the present study (for only 1/4 of the patients). Lacrimal gland acini were absent in all the patients in Weatherhead’s series [1]. The associated lacrimal tissue is smaller in accessory lacrimal gland lesions than in main LG lesions. Moreover, glandular acinar tissue undergoes progressive atrophy during dacryops formation [1, 5]. This can be missed without meticulous tissue sampling and sectioning. Additionally, meticulous surgical separation of the cyst from any surrounding tissues, including lacrimal glandular tissue, may explain the lack of associated lacrimal tissue.

A microscopic serpiginous configuration of cyst cavities was present in most patients (43/48) in our series. This finding was highlighted by Jakobiec et al. [5] and was present in 12/14 of their patients.

In the present study, the mean age at presentation was 28.5 years (range 3–66). In previously reported cases of accessory lacrimal gland ductal cysts, the mean age at disease onset was 30.5 years (range 2–81). Reports including main LG dacryops preponderance showed a higher mean age ranging from 48 to 52 years, based on a review of the literature by Fung et al. [16].

A female predilection was reported by Shield et al. [20] (75%), Alsarhani et al. [11] (72.7%) and Betharia et al. [8] (100%). Similarly, there was a female prevalence (64.6%) in our study. This can be attributed to the fact that trachomatous scarring, the most frequent etiological association of WGDCs in this study, occurs at higher rates in women [21].

WGDCs can be diagnostically challenging with a possibility of misdiagnosis [5, 11].

Clinical clues aiding in the diagnosis include the cyst’s subconjunctival location, adherence to the nonmarginal tarsal border, immobility, lack of tenderness, and positive transillumination. These lesions more frequently appear in the upper eyelid medially or centromedially, likely because more WGs exist in this location [1, 3, 11, 19]. WGDCs tend to grow insidiously; however, a rapid increase in size may occur and this should raise the suspicion of cyst complications such as hemorrhage or infection [19]. A transverse fibrotic band along the nonmarginal tarsal border in the area of the cyst was noted in all patients, independent of the eyelid affected. These fibrotic changes were different from those observed in palpebral conjunctival scarring or in Arlt’s line, which is pathognomonic for trachoma. This finding was observed in the photographs in previous reports and may have been overlooked [2, 3, 16]. It is unclear whether this finding represents a sequela of previous conjunctival inflammation that leads to obstruction of several adjacent duct orifices contributing to dacryops formation or whether it is simply a reactive response to fluid leakage from the cysts.

Distinguishing WGDCs from other cysts in the eyelid or anterior orbit can pose diagnostic complexities, both clinically and histologically. Krause gland ductal cysts exhibit similar characteristics; however, they are anatomically located within the fornices and lack fixation to the tarsal plate [22]. Another condition that is frequently confused with WGDC is conjunctival inclusion cyst, which results from the entrapment of conjunctival epithelial tissue within the substantia propria. These cysts are typically lined by either a double layer of low cuboidal cells or, more commonly, multilaminar nonkeratinizing squamous cells [5, 11, 23]. Absence of history of trauma or surgery, fixation to the tarsal plate, serpiginous microscopic cyst configuration, and presence of bilaminar cuboidal cells, rather than multilaminar nonkeratinizing squamous cells, are indicative features of WGDCs rather than conjunctival inclusion cysts [5, 11].

Various treatment modalities for dacryops have been described. Generally, complete excision through a conjunctival cul-de-sac route has been accepted as the treatment of choice for accessory lacrimal gland ductal cysts [1, 3]. However, this method is associated with more adverse events than the transcutaneous approach in our series. Intraoperative cyst rupture was greater with the transconjunctival route. The scarred conjunctiva restricts lid eversion of the eyelid, particularly in the upper eyelid, with poor cyst exposure. Additionally, everted lids force thin-walled cysts under tension and increase the risk of rupture. In contrast, the transcutaneous approach allows wider exposure of the cyst. Dissection can be performed easily away from the area of fibrotic adhesions and tarsal attachment where cyst rupture usually occurs.

Intraoperative cyst rupture did not significantly increase the risk of cyst recurrence in the study groups. Nevertheless, early cyst rupture with wall collapse during surgery poses challenges in identifying the cyst capsule through the conjunctiva, increasing the likelihood of incomplete excision of the cyst wall and the potential for recurrence [24].

An incision through the scarred conjunctiva leads to a large gap with an increased risk of tissue loss, making primary closure difficult. Therefore, conjunctival grafting is more frequently needed with this approach.

Postoperative ptosis was significantly greater in patients treated via the transconjunctival route. A direct conjunctival incision may trigger conjunctival inflammation and further scarring and shortening of the fornix, increasing the risk of mechanical ptosis. In contrast, the skin approach allows exploration of the levator aponeurosis and repair of dehiscence, if any [1].

While the higher detection of orbital signs in the transconjunctival group might suggest more advanced forms of disease, intraoperative inspection of the cysts showed no orbital extension. This indicates that the detected orbital signs were not necessarily due to advanced disease. This is further supported by the lack of significant difference between the two groups in the intraoperative measurements of the cyst.

Less invasive surgical methods, including simple aspiration, needling and marsupialization, have been used [12, 13]. Cysts readily refill after simple aspiration. Similarly, we feel that needling is an inappropriate procedure, and only one reported case was treated with needling [12]. Six of our patients had recurrent cysts following aspiration or needling performed before being referred to our institution.

Marsupialization entails deroofing of the cyst wall and is a less invasive treatment. The procedure does not require meticulous dissection of the cyst wall, which is technically trickier and more time consuming [13]. However, this approach may lead to recurrence with additional scarring in the future.

### Limitations

The retrospective nature of the study, nonrandomization, and small sample size are notable limitations to the present study. In addition, there may have been some bias with regards to transcutaneous versus transconjunctival approach selection by the surgeon. The authors historically utilized the transconjunctival approach for removing the WGDCs but have progressively switched to the transcutaneous approach in recent years. This shift was prompted by observing a notable occurrence of intraoperative cyst rupture, postoperative ptosis, and chronic eye irritation associated with conjunctival grafting associated with the transconjunctival route. Due to insufficient data on patients treated with the transconjunctival approach in earlier times, they were excluded from the study. This explains the relatively smaller number of patients and longer duration of follow-up among those treated with this approach.

## Conclusions

This article presents the largest series of WGDCs, highlighting the clinicopathologic characteristics and results of surgical removal. The data herein suggests that the transcutaneous approach has more favorable outcomes compared to the conjunctival route.

### Electronic supplementary material

Below is the link to the electronic supplementary material.

Supplementary Material 1

Supplementary Material 2

Supplementary Material 3

Supplementary Material 4

## Data Availability

The data that support the findings of this study are available from the corresponding author with the permission of Fayoum University Hospitals upon reasonable request.
