# Supplementary material for: Clinical characteristics and surgical outcomes of transcutaneous versus transconjunctival excision of Wolfring gland ductal cysts
Source: BMC Ophthalmol. 2024 Apr 16;24:164. doi: 10.1186/s12886-024-03420-x (PMC11020823; doi:10.1186/s12886-024-03420-x)
Supplement: Supplementary file 1 — Supplementary Material 1 [file 12886_2024_3420_MOESM1_ESM.pptx]

## Slide 1
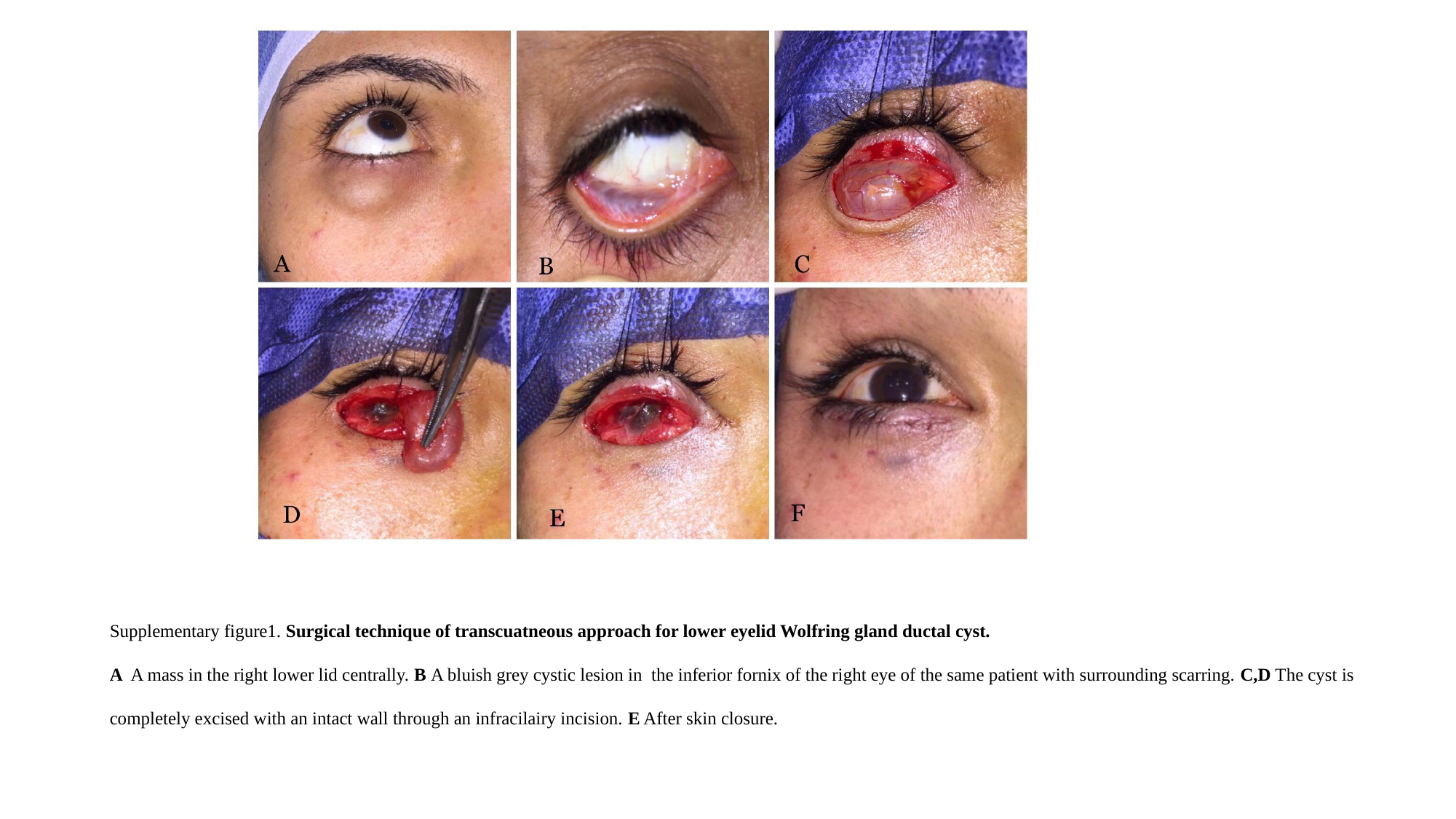

# Supplementary figure1. Surgical technique of transcuatneous approach for lower eyelid Wolfring gland ductal cyst. A A mass in the right lower lid centrally. B A bluish grey cystic lesion in the inferior fornix of the right eye of the same patient with surrounding scarring. C,D The cyst is completely excised with an intact wall through an infracilairy incision. E After skin closure.
