# Supplementary material for: Clinical characteristics and surgical outcomes of transcutaneous versus transconjunctival excision of Wolfring gland ductal cysts
Source: BMC Ophthalmol. 2024 Apr 16;24:164. doi: 10.1186/s12886-024-03420-x (PMC11020823; doi:10.1186/s12886-024-03420-x)
Supplement: Supplementary file 2 — Supplementary Material 2 [file 12886_2024_3420_MOESM2_ESM.pptx]

## Slide 1
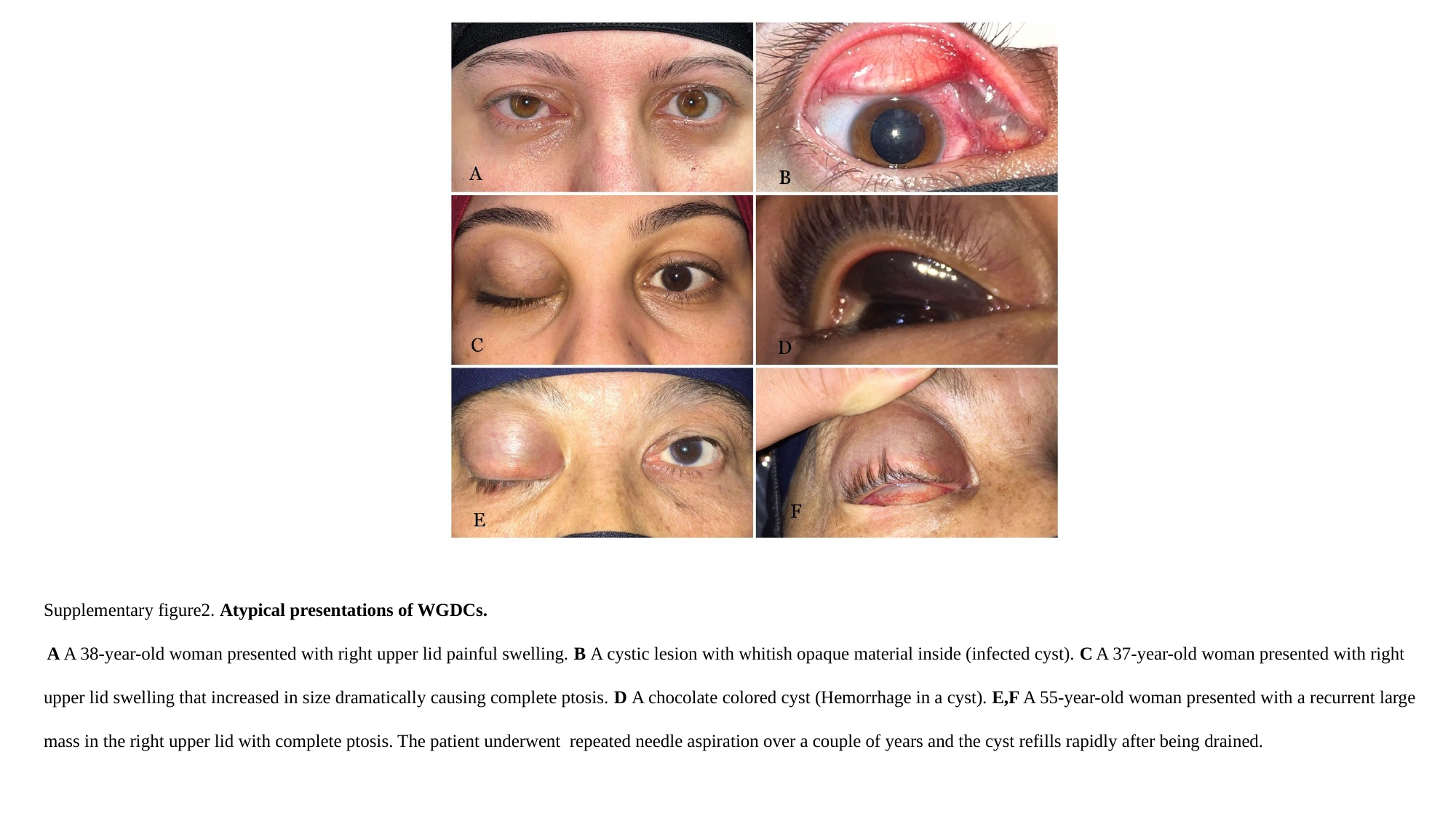

# Supplementary figure2. Atypical presentations of WGDCs. A A 38-year-old woman presented with right upper lid painful swelling. B A cystic lesion with whitish opaque material inside (infected cyst). C A 37-year-old woman presented with right upper lid swelling that increased in size dramatically causing complete ptosis. D A chocolate colored cyst (Hemorrhage in a cyst). E,F A 55-year-old woman presented with a recurrent large mass in the right upper lid with complete ptosis. The patient underwent repeated needle aspiration over a couple of years and the cyst refills rapidly after being drained.
