# Supplementary material for: Clinical characteristics and surgical outcomes of transcutaneous versus transconjunctival excision of Wolfring gland ductal cysts
Source: BMC Ophthalmol. 2024 Apr 16;24:164. doi: 10.1186/s12886-024-03420-x (PMC11020823; doi:10.1186/s12886-024-03420-x)
Supplement: Supplementary file 3 — Supplementary Material 3 [file 12886_2024_3420_MOESM3_ESM.pptx]

## Slide 1
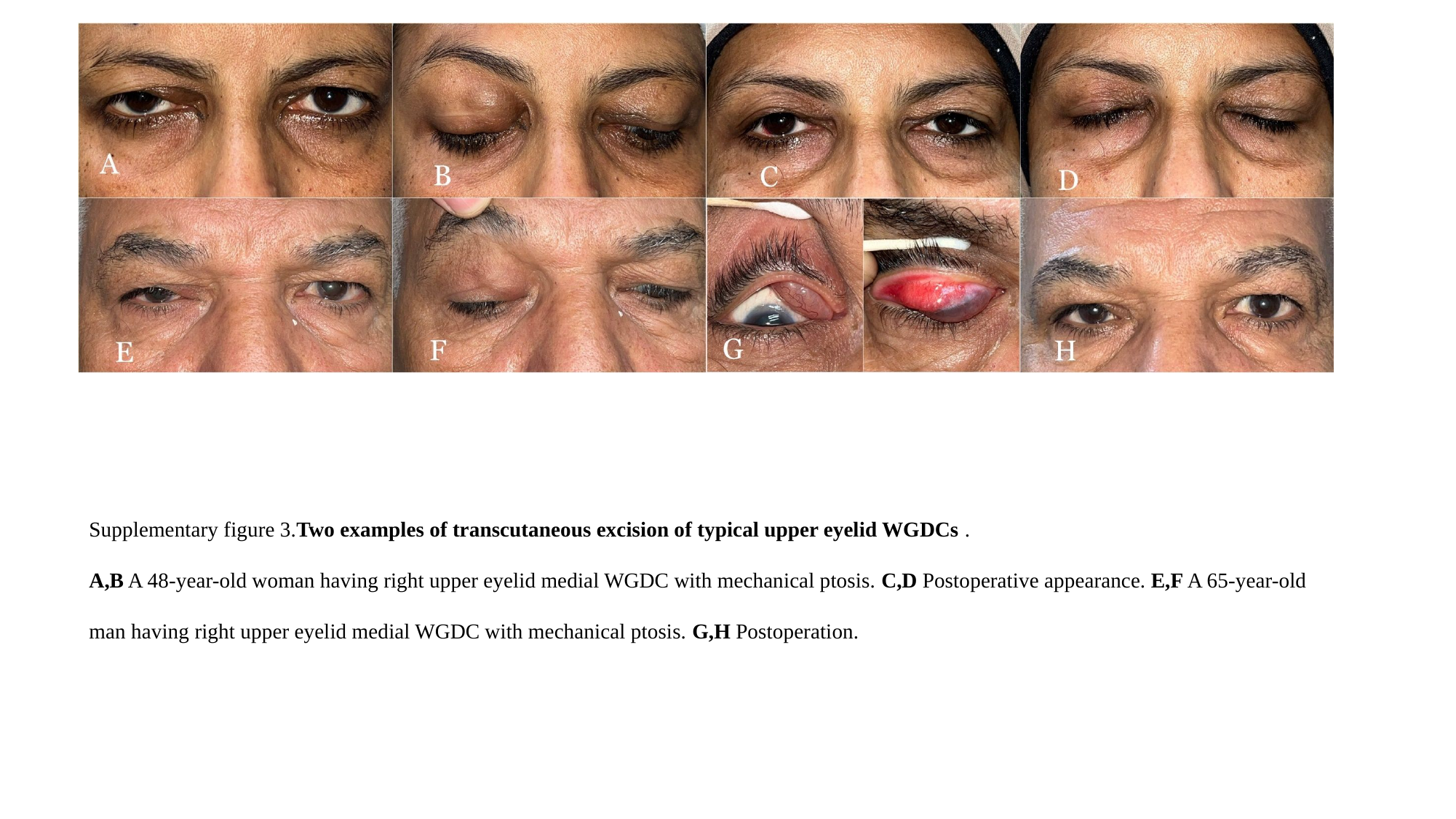

# Supplementary figure 3.Two examples of transcutaneous excision of typical upper eyelid WGDCs . A,B A 48-year-old woman having right upper eyelid medial WGDC with mechanical ptosis. C,D Postoperative appearance. E,F A 65-year-old man having right upper eyelid medial WGDC with mechanical ptosis. G,H Postoperation.
