# Supplementary material for: Clinical characteristics and surgical outcomes of transcutaneous versus transconjunctival excision of Wolfring gland ductal cysts
Source: BMC Ophthalmol. 2024 Apr 16;24:164. doi: 10.1186/s12886-024-03420-x (PMC11020823; doi:10.1186/s12886-024-03420-x)
Supplement: Supplementary file 4 — Supplementary Material 4 [file 12886_2024_3420_MOESM4_ESM.pptx]

## Slide 1
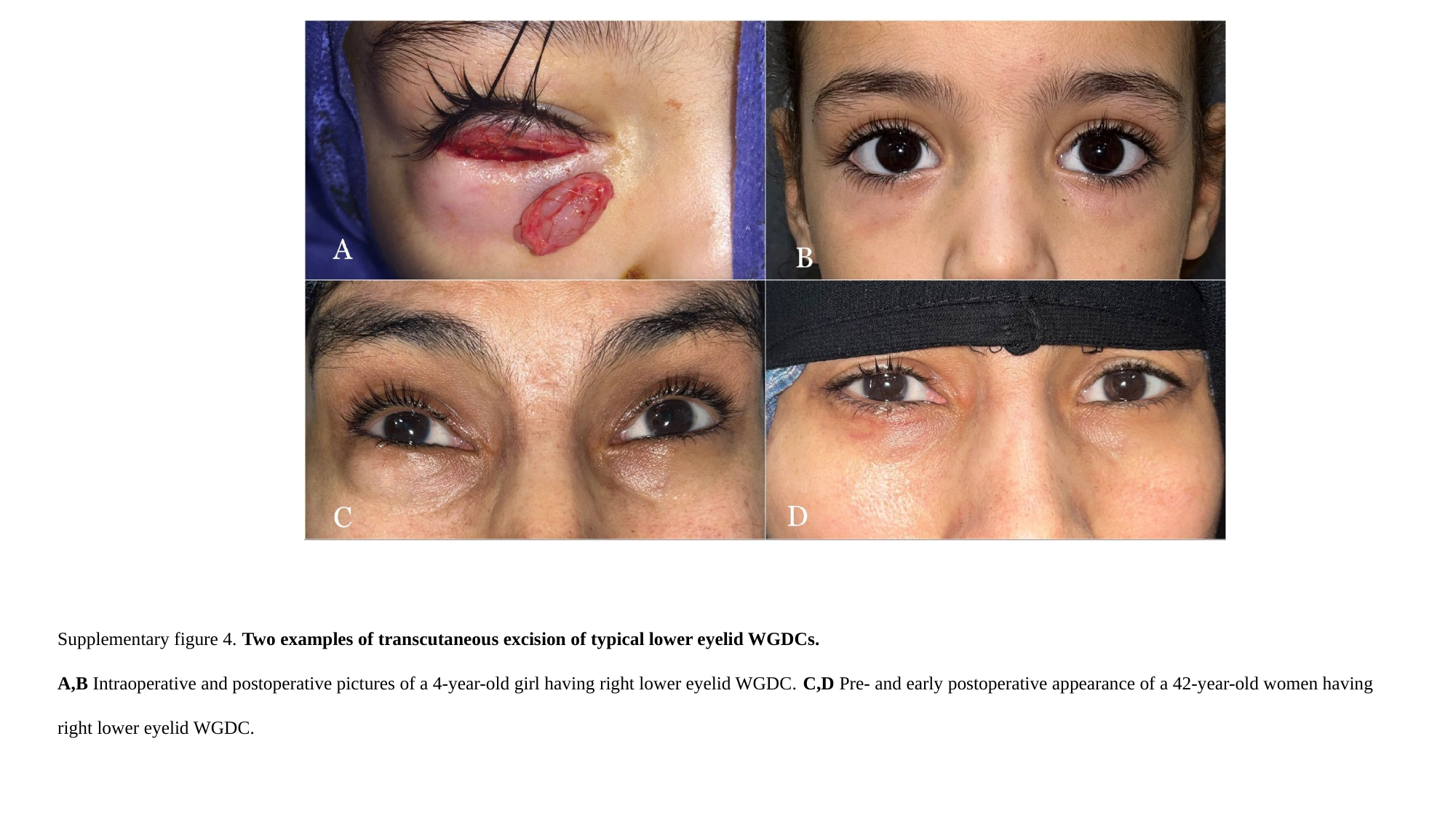

# Supplementary figure 4. Two examples of transcutaneous excision of typical lower eyelid WGDCs. A,B Intraoperative and postoperative pictures of a 4-year-old girl having right lower eyelid WGDC. C,D Pre- and early postoperative appearance of a 42-year-old women having right lower eyelid WGDC.
